# The reference human nuclear mitochondrial sequences compilation validated and implemented on the UCSC genome browser

**DOI:** 10.1186/1471-2164-12-517

**Published:** 2011-10-20

**Authors:** Domenico Simone, Francesco Maria Calabrese, Martin Lang, Giuseppe Gasparre, Marcella Attimonelli

**Affiliations:** 1 Dipartimento di Biochimica e Biologia Molecolare 'E. Quagliariello', Università di Bari, Bari 70126, Italy; 2 Dipartimento di Scienze Ginecologiche, Ostetriche e Pediatriche, U.O. Genetica Medica, Università di Bologna, Bologna 40138, Italy

## Abstract

**Background:**

Eukaryotic nuclear genomes contain fragments of mitochondrial DNA called NumtS (Nuclear mitochondrial Sequences), whose mode and time of insertion, as well as their functional/structural role within the genome are debated issues. Insertion sites match with chromosomal breaks, revealing that micro-deletions usually occurring at non-homologous end joining *loci *become reduced in presence of NumtS. Some NumtS are involved in recombination events leading to fragment duplication. Moreover, NumtS are polymorphic, a feature that renders them candidates as population markers. Finally, they are a cause of contamination during human mtDNA sequencing, leading to the generation of false heteroplasmies.

**Results:**

Here we present RHNumtS.2, the most exhaustive human NumtSome catalogue annotating 585 NumtS, 97% of which were here validated in a European individual and in HapMap samples. The NumtS complete dataset and related features have been made available at the UCSC Genome Browser. The produced sequences have been submitted to INSDC databases. The implementation of the RHNumtS.2 tracks within the UCSC Genome Browser has been carried out with the aim to facilitate browsing of the NumtS tracks to be exploited in a wide range of research applications.

**Conclusions:**

We aimed at providing the scientific community with the most exhaustive overview on the human NumtSome, a resource whose aim is to support several research applications, such as studies concerning human structural variation, diversity, and disease, as well as the detection of false heteroplasmic mtDNA variants. Upon implementation of the NumtS tracks, the application of the BLAT program on the UCSC Genome Browser has now become an additional tool to check for heteroplasmic artefacts, supported by data available through the NumtS tracks.

## Background

Human mitochondrial DNA (mtDNA) is widely used for phylogenetic, forensic and clinical studies and many features like maternal inheritance, absence of recombination and lack of efficient repair systems are well known and extensively studied. Recent advances in genetics provide researchers with mitochondrial DNA sequences located within the nuclear genome, thus allowing the investigation of intriguing aspects of genome organization. Fragments of mtDNA that give rise to nuclear mitochondrial sequences (NumtS) are found in many eukaryotic nuclear genomes and believed to derive from damaged mitochondria [1]. The discovery of these genomic elements dates back to 1967 through hybridization experiments on mouse liver between mtDNA and nuclear genome [2]. NumtS generation may have started soon after the endosymbiontic event [3] although the underlying mechanisms are still unclear and time and mode of arrival from mitochondria to nucleus have not been defined. As far as the mode, the most credited hypothesis suggests that in presence of mutagenic agents or under stress conditions, fragments of mtDNA may escape the organelles, reach the nucleus and likely insert into nuclear DNA during double-strand breaks (DSB) repair by the non-homologous end joining (NHEJ) machinery, although other mechanisms have been proposed [4,5]. It is commonly accepted that most human NumtS have originated before modern man, although evidences of NumtS recent insertions as well as their duplication in human genomes have been reported [6-15]. As a consequence, some NumtS display a highly polymorphic behaviour, as they can occur in homo- or heterozygosis, or be absent in different individuals at specific loci. These features render them candidates as population markers, as already suggested [16].

**Figure 1:**
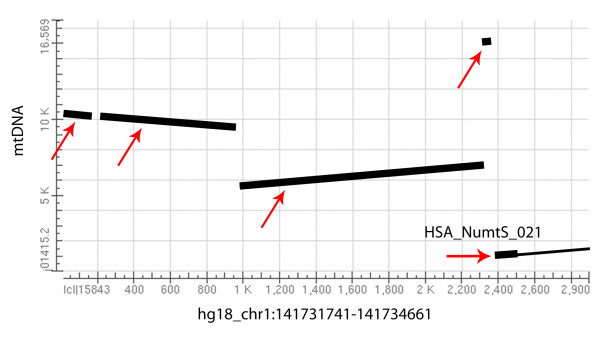
**BLAST2seq dotplot output**. The dotplot shows the results obtained by applying BLAST2seq between rCRS and chromosome 1 region containing HSA_NumtS_021 (release RHNumtS.1). The thin line refers to the part of the HSP_NumtS_021 already annotated in the RHNumtS.1 compilation. NumtS added in RHNumtS.2 are marked with thick lines and highlighted by red arrows.

Nonetheless, several criteria must be fulfilled before human NumtS can ascend to the status of evolutionary markers. NumtS identification, quantification and mapping must be completed and refined in the attempt to finally define the asset of mitochondrial sequences within the human nuclear genome, *i.e*. the human NumtSome. Indeed, despite scattered data on NumtS evolution and mechanisms of insertions being published [10,11,13], these results are based on pools of raw data that are not readily available. Moreover, it must be considered that accurate mapping is highly dependent on the quality of the genome assembly for each organism and on algorithms and parameters applied *in silico*. The need to provide a well-characterized, exhaustive database containing the human NumtSome has led us to generate the Reference Human NumtS compilation (RHNumtS), based on i) database similarity searching programs where the human mtDNA reference sequence (rCRS [17]) is compared to the human nuclear genome sequences (build hg18 available both at NCBI and UCSC sites) and ii) the comparison with other already published compilations [1,5,10,13]. Because of a great discrepancy among data obtained upon implementation of different protocols, stringent parameters were utilized during the production of the first release, RHNumtS.1 [18]. However in the process of *in vitro *validation, of the first release, evidences of additional mitochondrial fragments (Figure 1) not reported in RHNumtS.1 prompted a continuous work of protocol revision that has led to an extended and deeply validated second release, RHNumtS.2. The RHNumtS.2 also includes revisited data from smaller, scattered previously published compilations [1,5,10,13,18]. Here we report the optimized protocol that allows the detection of human NumtS. Upon a thorough validation by amplification and sequencing of the human NumtSome, here completed for the first time, both nuclear and mitochondrial human NumtS tracks have been created and made available through the UCSC Genome Browser.

## Results

### The RHNumtS.2 compilation

The RHNumtS.2 compilation was obtained through an *in silico *hybridization between each human chromosome (build hg18) and the reference human mitochondrial genome rCRS [GenBank:NC_012920]. The process returned 766 High Scoring Pairs (HSPs), *i.e*. mitochondrial fragments similar to nuclear sequences, hereafter named HSP_NumtS, whose alignment lengths ranged from 31 to 14904 bp. The similarity percentage of each fragment versus the rCRS sequence ranged from 63% to 100%. HSP_NumtS showing evident neighbourhood on both nuclear and mitochondrial genomes were merged in a single NumtS (assembled NumtS) according to the criteria described in the Methods section. NumtS covering the D-loop region were returned by BLASTN as different HSPs, as in the rCRS EMBL/GenBank/DDBJ databank entry the D-loop is split into the end (positions 16024-16569) of the sequence followed by the start (positions 1-576). Therefore, HSP_NumtS close in the nuclear genome and mapping on the D-loop region, were also merged in a single assembled NumtS: this device fitted our joining protocol to the circularity of the mitochondrial genome. Overall, RHNumtS.2 annotates 766 human HSP_NumtS corresponding to 585 assembled Human NumtS inclusive of the 190 annotated in RHNumtS.1 [18]. Covered genome amounts to 627410 bases. The complete RHNumtS.2 compilation is reported here in the additional file 1 RHNumtS.2.xls. A NumtS ID was assigned to each assembled NumtS with a format HSA_NumtS_xxx, where HSA stands for *H. sapiens *and xxx is a three-digit code.

**Figure 2:**
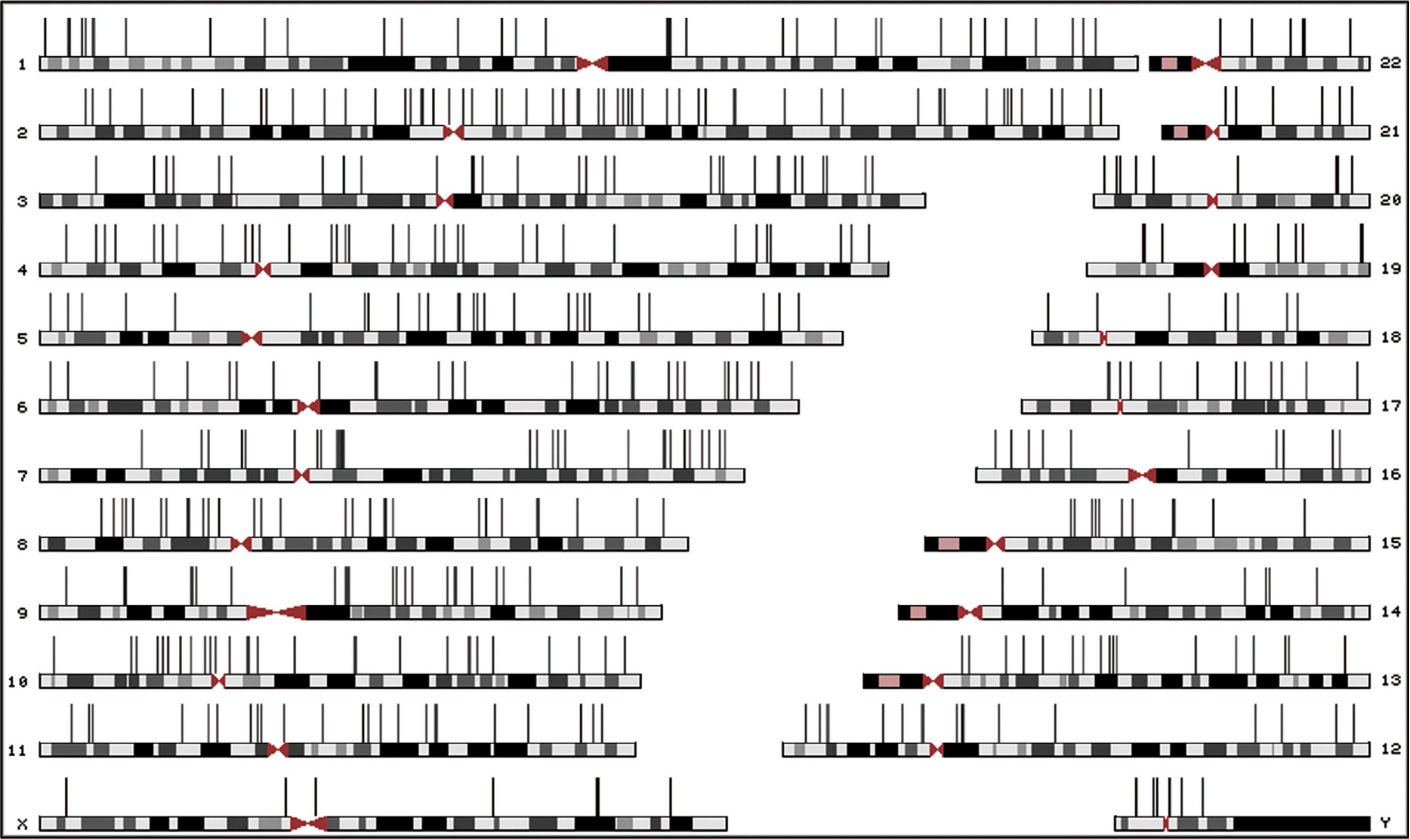
**Mapping of the RHNumtS elements on the human karyotype**. Vertical bars refer to the NumtS location. NumtS that are close together merge at this zoom level.

**Figure 3:**
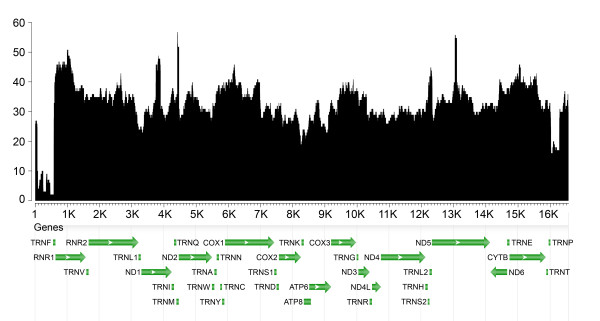
**Coverage of human mtDNA sites on the whole human nuclear genome**. Different genes are marked as follows: ATP6 and ATP8, ATPase subunits 6 and 8; COXI, COXII, COXIII, cytochrome c oxidase subunits I, II, III; CYTB, cytochrome b; ND1-6 and 4L, NADH dehydrogenase subunits; tRNA genes are indicated according to the transported amino acid. L1, Leu (UUR); L2, Leu (CUN); S1, Ser (UCN); S2, Ser (AGY). Horizontal axis = nucleotide position on mitochondrial genome; vertical axis = number of occurrences in the nuclear genome.

Detailed statistics on the NumtS length and similarity distributions are shown in Table 1. Mapping of NumtS (Figure 2) showed that chromosome 2 and chromosome 18 are respectively the most and least densely populated by this class of genomic elements in terms of base pairs overall length (Table 2). A statistical correlation showed that there is a linear relationship between the number of NumtS located on each chromosome and the relative chromosome length (Pearson's *r *= 0.82). On the other hand, the value obtained by correlating the number of NumtS for each chromosome and the genic density (0.22) suggests that, at chromosome level, there is no evidence of NumtS distribution biased by the presence of genes. NumtS mtDNA coverage (Figure 3) highlights that the fragments add up to several copies of the mitochondrial genome. Over-represented regions encompass many tRNAs, the two ribosomal genes 12S and 16S, large portions of COX1, COX3, CYTB genes and short fragments of ND1, ND5 and ND6 genes. On the other hand, D-loop region, the whole ATP8 gene and most of ND1, ND4 and ND6 genes are under-represented.

**Table 1:** HSPs statistics concerning the Blast2seq application of the rCRS sequence (J01415)

	HSP similarities	HSP lengths (% of mt genome)	ΔHSP-span differences	Distances between concatenated HSPs
**min**	63.52	31 (0.1)	0	-8
**1st quartile**	73.74	94 (0.5)	0	215.25
**median**	78.41	215 (1)	1	320
**3rd quartile**	84.62	619 (3)	5	585.75
**max**	100	14836 (89)	160	6129

For each HSP, the ΔHSP-span difference is the absolute value of the difference between the HSP length on nuclear genome and the HSP length on the mitochondrial genome. The distance of -8 bp reported indicates overlapping of two concatenated HSPs on nuclear genome.

**Table 2:** NumtS number and percentage for each chromosome

Chr name	NumtS per Chr	Chr length (bp)	Total NumtS span (bp)	NumtS bp % per Chr
1	46	247249719	57670	0.023
2	92	242951149	108317	0.045
3	39	199501827	24203	0.012
4	38	191273063	36179	0.019
5	24	180857866	40163	0.022
6	31	170899992	11708	0.007
7	35	158821424	51356	0.032
8	37	146274826	46805	0.032
9	28	140273252	36854	0.026
10	33	135374737	21445	0.016
11	28	134452384	26640	0.020
12	40	132349534	8551	0.006
13	19	114142980	11159	0.010
14	8	106368585	8991	0.008
15	12	100338915	9002	0.009
16	11	88827254	17205	0.019
17	17	78774742	24285	0.031
18	7	76117153	1340	0.002
19	15	63811651	19373	0.030
20	10	62435964	5905	0.009
21	8	46944323	7210	0.015
22	8	49691432	9025	0.018
X	13	154913754	30845	0.020
Y	12	57772954	11510	0.020

Chromosome 2 displays the highest bp percentage of NumtS, while chromosome 18 the lowest. Chr: chromosome.

### Bench and *in silico *validation of RHNumtS.2

The *in silico *hybridization was based on a consensus human genome (build hg18) derived from the DNA sequencing from 6 different samples. Due to the polymorphic nature of human NumtS [13] and to the technical difficulties posed by repetitive sequences during assembly of the consensus genome, we proceeded to validate RHNumtS.2 on an individual of European origin, through amplification and sequencing (bench validation). HSP_NumtS not present in the European sample (HSA_NumtS_009, HSA_NumtS_426, HSA_NumtS_522) were validated in an Ethiopian sample. Additionally, *in silico *validation was carried out on genomic annotations from eight HapMap samples.

**Figure 4:**
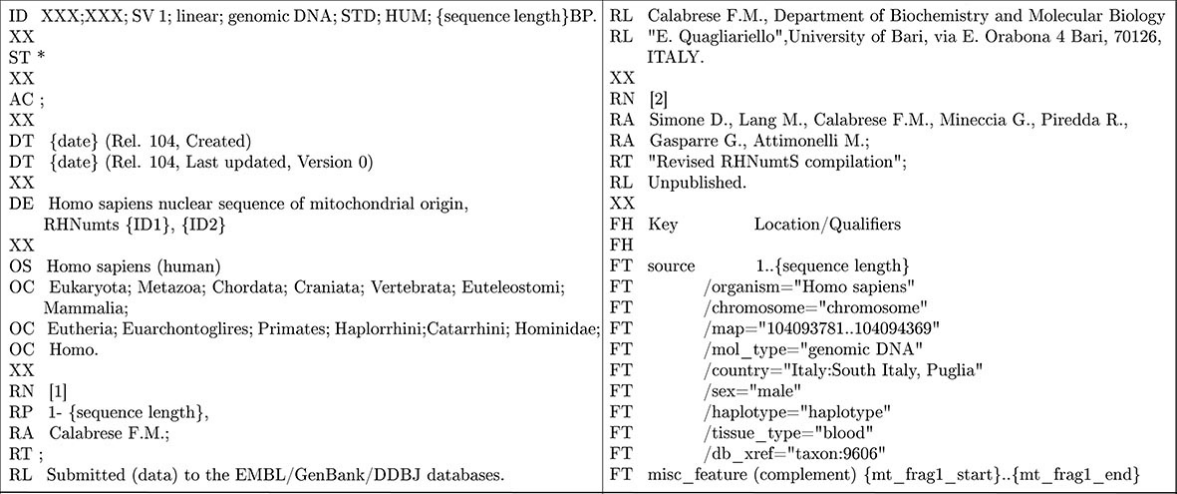
**EMBL flat-file template**. The template was designed for the WebIN submission of the sequenced NumtS.

Design of 355 specific primer pairs was performed, providing amplicons of molecular weight (MW) expected in presence of the NumtS, as confirmed by gel electrophoresis, allowing the validation of 354 HSP_NumtS. Overall 339/354 were successfully sequenced on both strands. Despite yielding an amplicon of expected MW in the case of NumtS presence, the remaining 15 PCR products contained long homopolymeric stretches or heterozygous repetitive satellite-like sequences, which rendered sequencing incomplete. Thus 44% of the total HSP_NumtS (339/766) were sequenced, which is the largest set of NumtS sequenced to date, to the best of our knowledge. Each obtained sequence was multi-aligned to the corresponding mtDNA fragment of the same individual, to the hg18 corresponding nuclear DNA fragment as well as to the rCRS mtDNA fragment. The multi-alignments are available in the additional file 2 Validated_NumtS_multial.txt. HSP_NumtS sequences were submitted to the EMBL databank through the WebIN submission tool (http://www.ebi.ac.uk/webin) available through the INSDC (International Nucleotide Sequence Database Collaboration) organization. To this purpose, a human NumtS EMBL flat-file format template was designed (Figure 4). The entry structure reports in the "Features" table the "misc_feature" data which allow the end-user accessing the INSDC databases to obtain the NumtS location on the nuclear genome and the mtDNA fragments of origin. The accession numbers are reported in additional file 3 NumtS_validation.xls. Overall, 279 assembled NumtS were validated with this approach (Table 3), *i.e*. those NumtS for which a specific primer pair could be designed.

**Table 3:** Validation statistics

Validation test	Validated Assembled NumtS (%)
PCR and HapMap	5 (0,85%)
PCR and Seq	26 (4,44%)
PCR, Seq and HapMap	247 (42,22%)
HapMap only	279 (47,69%)
PCR only	1 (0,17%)
Not validated	27 (4,62%)
Total	585 (100%)

Number and percentage of validated assembled NumtS according to the three different validation tests: PCR, sequencing and HapMap FES data analysis.

To further improve and complete the RHNumtS.2 validation, we compared the NumtS map versus the individual Fosmid End Sequences (FES) annotations related to eight HapMap samples. Table 4 reports the number of FES positive to the presence of NumtS and the number of NumtS mapped in the library of each individual. The 558 NumtS mapped on the FES are listed in additional file 3 NumtS_validation.xls. As a further confirmation, 252 of our 279 bench-validated NumtS were also retrieved from FES analysis. The presence of 27 NumtS (5%) could not be investigated and ascertained. Overall, 95.4% of the RHNumtS.2 was hence validated.

**Table 4:** *In silico *validation on HapMap FES data

HapMap identifier	Geographical origin	FES with NumtS	Validated NumtS
NA18517 (ABC7)	Yoruba	417	202
NA18507 (ABC8)	Yoruba	709	307
NA18956 (ABC9)	Japan	494	210
NA19240 (ABC10)	Yoruba	480	214
NA18555 (ABC11)	China	490	228
NA12878 (ABC12)	CEPH	533	229
NA19129 (ABC13)	Yoruba	504	230
NA12156 (ABC14)	CEPH	574	245

For each individual, discrepancy between the number of FES with NumtS and the corresponding number of validated NumtS is due to the different coverage of NumtS loci in FES.

### The UCSC human NumtS tracks

In order to facilitate browsing of NumtS sequences, we implemented human NumtS tracks using the UCSC Genome Browser tools upon mapping on the hg18 build. Four different NumtS tracks were designed and implemented under the section 'Variation and Repeats'.

**Figure 5:**
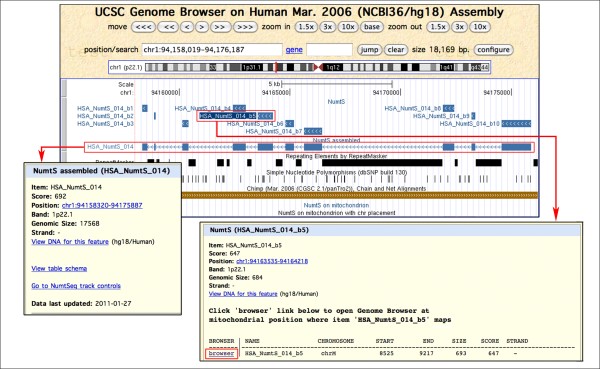
**NumtS UCSC Genome Browser tracks (I): nuclear tracks**. NumtS browsing on UCSC Genome Browser can be performed by displaying a genomic region of interest or by directly searching a NumtS ID in the Genome Browser gateway. The graph shows the genomic region where the HSA_NumtS_014 is located (in the 'NumtS assembled' track) and the ten constituent HSP_NumtS (in the 'NumtS' track). Details about each item can be displayed by clicking on it, as pointed out by the red arrows. The red-framed "browser" link in the HSA_NumtS_014_b5 detail page links to the mitochondrial NumtS tracks shown in Figure 6. An essential flowchart describing NumtS tracks browsing is given in additional file 4 UCSC_NumtS_browsing.pdf.

**Figure 6:**
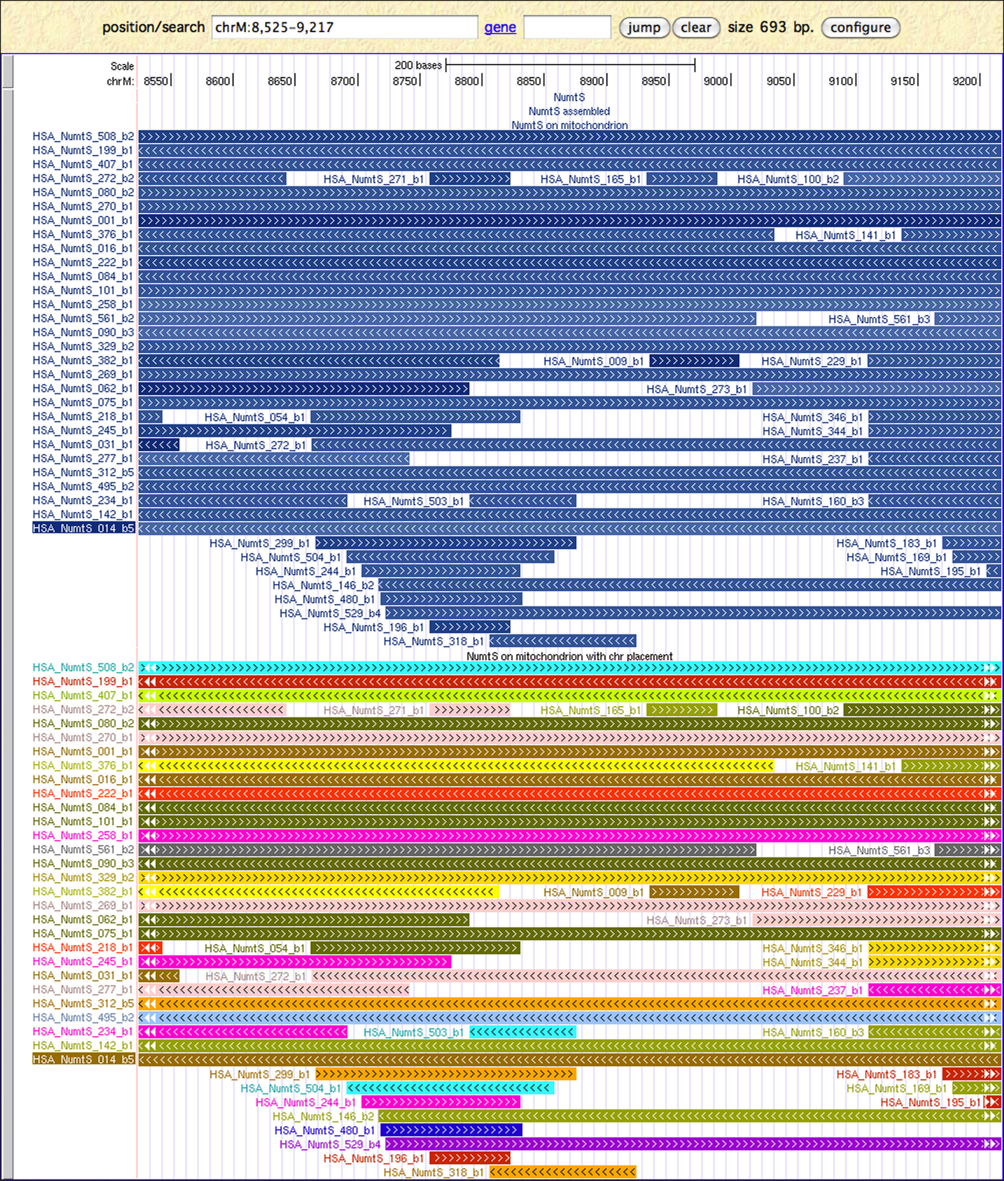
**NumtS UCSC Genome Browser tracks (II): mitochondrial tracks**. The mitochondrial genome region where HSA_NumtS_014_b5 maps is displayed, and all the HSP_NumtS mapping entirely or partially on the same region are reported within the 'NumtS on mitochondrion' track and the 'NumtS on mitochondrion with chromosome placement' track. The HSA_NumtS_014_b5 ID is highlighted in both tracks. An essential flowchart describing NumtS tracks browsing is given in additional file 4 UCSC_NumtS_browsing.pdf.

The **'**NumtS' track shows the mapping of the HSPs returned by BLASTN on the nuclear genome, namely the HSP_NumtS. Items shading reflects the similarity score obtained by BLASTN and the arrows direction is concordant with the strand on which the NumtS is aligned. A link to mtDNA mapping is also provided. The 'NumtS assembled' track shows items obtained by assembling HSPs annotated in the 'NumtS' track fulfilling the conditions described in the Methods section. The 'NumtS on mitochondrion' track shows mapping of the HSP_NumtS on the mitochondrial genome. Items shading reflects the similarity returned by BLASTN, and the direction of the arrows is concordant with the alignment strand. For every item, a link pointing to the nuclear mapping is provided. The 'NumtS on mitochondrion with chromosome placement' track shows the mapping of the HSP_NumtS on the mitochondrial genome, but the items are depicted according to the colours assigned to each human chromosome by the UCSC Genome Browser. For every item, a link pointing to the nuclear mapping is available. The four NumtS tracks annotate the entire RHNumtS.2 compilation. A screenshot example of the browsing of the UCSC NumtS tracks is reported in Figure 5 and 6, while a brief explanation of their surveying is provided in additional file 4 UCSC_NumtS_browsing.pdf.

## Discussion

The number of NumtS reported in this release, RHNumtS.2, has increased about by three fold over the first one [18], while the amount of bases covered has increased about by 1.6 fold. More details about the extension of the previous release are provided in the additional file 1 RHNumtS.2.xls (sheet "RHNumtS.1_extension"). RHNumtS.2 includes all the NumtS annotated in RHNumtS.1; 79 of them weren't extended at all, while the median value of the extension ratio is 1.05. For ten NumtS, the extension was quite considerable (extension ratio > 6). Indeed the protocol designed for the production of the first release was aimed to produce a reference compilation, *i.e*. a collection of sequences located on the reference human genome build and showing strong evidences allowing to define them as "NumtS". The less stringent protocol here applied has allowed us to recognize 585 NumtS, over 95% of which have been validated here for the first time, either by bench approaches (PCR and sequencing) or *in silico *on eight samples collected within the international HapMap project.

**Figure 7:**
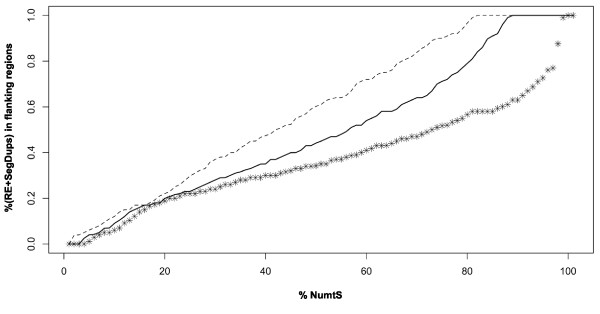
**Cumulative distributions of NumtS flanking regions repetitive elements**. The cumulative distribution of repetitive elements (RE) and segmental duplications (SegDups) content in the 1000 bp 5' and 3' flanking regions of NumtS is reported as calculated on: a) the whole collection of NumtS; b) the PCR-validated NumtS; c) the NumtS not validated by PCR. The graph shows that 60% of the PCR-validated NumtS contained less than 41% RE and SegDups in their flanking regions, while this percentage increased (72%) when considering NumtS not validated by PCR.

The novelty here presented is the implementation of the NumtS annotations in the UCSC Genome Browser. The "NumtS Sequence" tracks are available in the hg18 release of the human genome, in the track section "Variation and Repeats": this feature broadens the examination potential of NumtS within their genomic context, as they can be intuitively displayed and merged with annotations of other genomic elements available in the Genome Browser. Browsing of the 1000 bp-long flanking regions of NumtS shows that about 97% of NumtS reside in loci associated to repeated elements of various types. This evidence led us to suppose that amplification might be difficult because of the risk to obtain non-specific amplification. Notwithstanding this evidence, 279 NumtS were amplified, 15% of which fell within highly repeated regions (Figure 7). As a complementary approach, FES analysis revealed to be informative with respect to the validation of *in silico *detected NumtS located within duplicated regions. As marked in Table 3 and annotated in additional file 3 NumtS_validation.xls (sheet "Non-validated NumtS"), only 27 NumtS have not been validated, *i.e*. those mainly involved in duplication events.

A result that it is worth commenting concerns the coverage of the mitochondrial genome provided by NumtS. mtDNA fragments related to tRNAs and extended portions of the two ribosomal genes as well as of COX1, COX3 and CYTB genes are over-represented, whereas the D-loop region and other genes such as ATP8, ND1, ND4 and ND6 were the least represented within the nuclear genome. These data are in agreement with the conservation degree of the mitochondrial genes in mammals reported in [19]. The diversified observed frequencies of mtDNA fragments could be justified by taking into account that the RHNumtS.2 compilation results from an *in silico *hybridization between the modern human mtDNA and the human nuclear genome, therefore more evolutionary conserved mitochondrial regions may have higher chances to be detected with such strategy. Further bioinformatics analyses based on different approaches could also contribute to recognize more ancestral events that had led mitochondrial fragments to insert into nuclear genome. Finally, with respect to the apparently unbalanced distribution of the NumtS on the different chromosomes, although preliminary evidence showed a preferential NumtS localization in non-coding regions, a more detailed analysis of each chromosome and NumtS is required to explain the insertion bias towards specific chromosomal regions.

## Conclusions

In this paper we present the most exhaustive overview on the human NumtSome implemented in the RHNumtS.2 compilation, a resource whose aim is to support different research applications ranging from studies concerning human structural variation, diversity, and disease, as well as the detection of false heteroplasmic mtDNA variants.

## Methods

### Blasting of the human nuclear genome versus the mitochondrial genome

BLAST2seq implements the BLASTN program (release 2.2.19 of the BLAST suite) [20] applied to the comparison between two sequences. The run was performed on a local server. Twenty-four runs were launched, one for each human chromosome sequence available through the hg18 build. The human mtDNA reference sequence rCRS [GenBank:NC_012920] was used as query. Scoring parameters were fixed as follows: 2 for match reward, -3 for mismatch penalty; -5 for gap opening, -2 for gap extension. The e-value was fixed to 1e-03. The hg18 chromosome sequences were fetched using the get-genome program of the GMAP package [21]. Each fragment of each chromosome aligned with the J01415.2 mtDNA whose e-value was lower than the fixed threshold produced an HSP (High Scoring Pair).

### NumtS assembling

The assembling of the HSPs was performed with spreadsheet interpolation and manual inspection, strongly supported by graphical display of the HSP_NumtS with a custom annotation tool available on the UCSC Genome Browser. HSP_NumtS located less than 2000 bp apart on a specific chromosome and corresponding to two mtDNA fragments, not more than 2000 bp apart and oriented in the same direction, were merged in a single NumtS and here named as 'assembled NumtS'. The fragment joining protocol was slightly modified for HSPs interposed by long repetitive elements (see HSA_NumtS_014 in Figure 5 for an example).

### Primer design

Primers were designed with primer BLAST software (http://www.ncbi.nlm.nih.gov/tools/primer-blast/) using as a template each NumtS extended by 1000 nucleotides from the 5' and 3' ends, and specifically locating primers in NumtS flanking regions. The NumtS sequences with their flanking regions were extracted from the UCSC Genome Browser (http://genome.ucsc.edu/) [22]. Primers were designed to ensure amplification, even in case of NumtS absence. For NumtS longer than 1200 bp, external/internal, internal/external, and/or internal/internal primer pairs were designed. To exclude co-amplification of mtDNA, primers were validated through BLAST analysis, by comparison with the mitochondrial sequence of the European individual [18]. Moreover, in order to avoid primers self-hybridization, the Oligo Analysis tool available at Operon web site (http://www.operon.com/technical/toolkit.aspxis), was used. Sequences of primer pairs are provided in additional file 3 NumtS_validation.xls (sheet "Validated_NumtS").

### PCR amplification and sequencing

The validation of RHNumtS.2 was carried out on DNA extracted from blood of an individual of European origin belonging to a typical European mitochondrial haplogroup H2b. NumtS were amplified and sequenced as previously described [18]. HSP_NumtS not present in the European sample were validated in an Ethiopian sample whose mtDNA belonged to the L0 haplogroup. NumtS sequences were submitted to the EMBL databank and hence to the three Nucleic Acids databases joint in the international collaboration INSDC. The WebIn tool available on-line was utilized (http://www.ebi.ac.uk/embl/Submission/). Accession numbers are provided in additional file 3 NumtS_validation.xls (sheet "Validated_NumtS").

### Multi-alignment

NumtS sequences were multialigned to the corresponding hg18 sequence inclusive of the NumtS flanking region and to the mtDNA fragments from rCRS and from the validation sample. The multi-alignment has been produced using the ClustalW program (http://www.ebi.ac.uk/Tools/ClustalW/) [23]. The whole data set of the multialigned NumtS is available in the additional file 2 Validated_NumtS_multial.txt. Multi-alignment of the sequences already published in [18] have been included in the dataset.

### Comparison of the RHNumtS.2 sequences versus the FES

The HapMap consortium [24] has made available 270 samples from Nigeria, China, Japan and North/West Europe. With the aim to study human structural variation, eight HapMap samples (Table 4) were selected and their genomic DNA was cloned using a fosmid subcloning strategy [25]. For each individual library the paired ends were sequenced and the obtained FES (Fosmid End Sequences) data were made publicly available (http://hgsv.washington.edu/).

The *in silico *validation of NumtS was based on a merging protocol carried out using the Galaxy package available at http://main.g2.bx.psu.edu/. For each sample, only clones with a single best concordant placement according to the FES-pair analysis previously described [25] were considered.

### NumtS tracks implementation

The NumtS tracks and the external links were produced starting from the RHNumtS.2 compilation spreadsheet, with manual manipulation and by using in-house shell and Python scripts. The human mitochondrial reference genome at the UCSC Genome Browser derives from an African individual [GenBank:NC_001807] and shows three insertions with respect to the rCRS. Therefore, the mitochondrial coordinates annotated in the additional file 1 RHNumtS.2.xls were re-mapped to NC_001807. The tracks have been released in bed format, one of the formats allowing the display of the tracks at the UCSC Genome Browser. Templates of the bed file format and the chromosome colour key are available on the UCSC Genome Browser help pages (http://genome.ucsc.edu).

## Authors' contributions

DS carried out the bioinformatics analyses aimed at extending the compilation, validating it by analysing the HapMap data and designed and implemented the UCSC tracks in collaboration with the UCSC staff. FMC designed primers for bench validation, produced the multialignments and submitted the produced sequences to INSDC; ML carried out the PCR amplification and the sequencing; GG and MA coordinated and supervised the whole project and wrote the manuscript. All authors read and approved the final manuscript.

## Supplementary Material

Additional file 1 **The RHNumtS.2 compilation**. The table reports 766 Human HSP_ NumtS fragments of which 150 have been assembled in a single NumtS according to the rules reported in the Results section. Each line refers to an HSP_NumtS and reports the **NumtS ID**, the **HSP_NumtS ID**, a code tracing the compilation updating procedure, the **chromosome **where the NumtS is located and the **strand **where BLAST mapped the NumtS, the positions on the **hg18 chromosome **and the **rCRS**. In the case of assembled NumtS, in the right hand side of the table, detailed information on each fragment is available. The last column reports the identity percentage as reported in the BLAST output for each HSP. Click here for file

Additional file 2 **Multi-alignments of sequenced NumtS**. This archive contains multi-alignments of sequenced NumtS with the corresponding nuclear and mitochondrial regions. Each file reports the multi-alignment of reference (hg18) NumtS sequence, the sequenced NumtS, the corresponding mitochondrial H2b sequence and the corresponding rCRS region. For the three NumtS not present in the European individual (HSA_NumtS_009, HSA_NumtS_426, HSA_NumtS_522), the sequence validated on the Ethiopian individual (L0) was multialigned with the reference (hg18) NumtS sequence and the sequence produced in the European sample; the gap present in the latter sequence matches with the nuclear region where the NumtS maps. Click here for file

Additional file 3 **The RHNumtS.2 Human NumtS validated by bench and/or *in silico *approaches**. The table reports two sheets, one for the validated NumtS and the other one for non-validated NumtS. In the sheet "Validated NumtS", for each validated NumtS the following data are reported: the NumtS ID code, the HSP_NumtS_ID, the accession number hold until published code referred to he INSDC submission, in the case when the NumtS has been sequenced, otherwise the flag 'not sequenced' is annotated, the flags (Y or N) indicating successful response to PCR, sequencing and *in silico *Hapmap sample validation; and finally the primer code and the forward and reverse primer sequences. In the sheet "Non-validated NumtS", the list of non-validated NumtS (neither by PCR/sequencing, nor by *in silico *approach) is provided, along with a possible explanation for validation failure. Click here for file

Additional file 4 **Browsing NumtS tracks on UCSC Genome Browser**. This file provides an essential guide to the browsing of the NumtS tracks available at UCSC Genome Browser. Click here for file
