# Supplementary material for: The reference human nuclear mitochondrial sequences compilation validated and implemented on the UCSC genome browser
Source: BMC Genomics. 2011 Oct 20;12:517. doi: 10.1186/1471-2164-12-517 (PMC3228558; doi:10.1186/1471-2164-12-517)
Supplement: Additional file 4 — Browsing NumtS tracks on UCSC Genome Browser. This file provides an essential guide to the browsing of the NumtS tracks available at UCSC Genome Browser. [file 1471-2164-12-517-S4.PDF]

Supplementary text

The Reference Human Nuclear Mitochondrial Sequences Compilation Validated and Implemented on the UCSC Genome Browser

Browsing NumtS tracks on the UCSC Genome Browser

Introduction

In this Supplementary Text we give an example of the browsing of the NumtS annotation tracks available at the UCSC Genome Browser. The aim of this document is to provide an essential flowchart to display NumtS in their nuclear and mitochondrial context, but the NumtS tracks can be also fully exploited upon application of the tools available at the UCSC Genome Browser (http://genome.ucsc.edu/) and in the Galaxy suite (http://main.g2.bx.psu.edu/). Following the steps described in this text, it is possible to display the same screens reported in Figures 5 and 6 of the main text, which describe the browsing of HSA_NumtS_014, an assembled NumtS made up of ten HSP_NumtS.

Activating NumtS tracks display

Go to the UCSC Genome Browser homepage (http://genome.ucsc.edu) and click on the "Genome Browser" link on the left. You will be redirected to the Genome Browser gateway. In the Mammal clade, select the genome "Human", assembly "Mar2006 (NCBI36/hg18)".

Starting points of NumtS tracks browsing can be

- entering a genome location of your interest

  or

- looking up for a NumtS of the RHNumtS.2 compilation.

In the first case, enter the chromosome location of HSA_NumtS_014 (chr1:94,158,019-94,176,187) in the "position or search term" window. NumtS tracks are not displayed in the Genome Browser by default, so you have to activate the "NumtS Sequence" tracks ("Variation and

Repeats" section) in "pack" mode. Refresh the webpage; HSA_NumtS_014 will be then displayed. In the second case, enter the NumtS code ID (HSA_NumtS_014) in the "position or search term" window. A result page will be displayed, showing that four tracks contain one or more items whose name contains the searched ID. The results reported suggest that HSA_NumtS_014 is an assembled NumtS made up of ten fragments, whose location is indicated by the results relative to the "NumtS" track and mitochondrial mapping by the results relative to the "NumtSMitochondrion" track. Selecting the item in the "NumtS assembled" track, you will be redirected to the Genome Browser, displaying the genomic region where HSA_NumtS_014 is located. Please note that all tracks returning at least one result from the NumtS ID text search are automatically activated. Activate the "Repeat Masker" track from the "Variation and Repeats" section and the "ChimpNet" track from the "Comparative Genomics" section.

Nuclear tracks: 'NumtS' and 'NumtS_assembled'

The NumtS track shows the assembled HSA_NumtS_014 and its ten components (Figure 5). The comparison with the Repeat Masker track highlights the presence of repeated elements among the blocks, explaining the fragmentation of a single mitochondrial insertion. The comparison with the "ChimpNet" track demonstrates that the region where HSA_NumtS_014 is located is syntenic between man and chimp, thus proving that the considered NumtS is ancestral to the time of origin of modern humans. By clicking on the item HSA_NumtS_014 (following the red arrow on the left in Figure 5), the NumtS assembled page description is opened. The following information can then be obtained: the similarity score for the assembled NumtS, the location on the genome, the chromosome band, the genomic fragment length and its orientation (strand + or – with respect to the alignment versus the mitochondrial genome) and finally the link for the download of the NumtS sequence. By clicking on the item HSA_NumtS_014_b5 (following the red arrow on the left in Figure 5), the NumtS page description is opened. Here, besides the information also available in the NumtS assembled page description, information concerning the mapping of the HSP_NumtS on the mitochondrial genome are reported and a link pointing to the mitochondrial genome position

where the NumtS is mapped is provided. By clicking on the 'browser' link (red framed in Figure 5) the mitochondrial track is displayed (Figure 6).

Mitochondrial tracks: 'NumtS on mitochondrion' and 'NumtS on mitochondrion with chromosome placement'

The mitochondrial genome region where HSA_NumtS_014_b5 maps is displayed (Figure 6), and all the HSP_NumtS mapping entirely or partially on the same region are reported within the "NumtS on mitochondrion" track and the "NumtS on mitochondrion with chromosome placement" track. The HSA_NumtS_014_b5 ID is highlighted in both tracks.

Note

If the flowchart described in this document is applied to the hg19 release of the human genome (by selecting the assembly "Feb. 2009 (GRCh37/hg19)" from the Genome gateway), the "NumtS on mitochondrion with chromosome placement" track will no longer be displayed, as it was substituted with the "Human NumtS on mitochondrion SNPs" track. This track shows the mapping of the HSPs on the mitochondrial genome, with the SNPs which fall within, derived from the comparison with the hg19 build.
